# The Percentage Administration of Chloroform

**Published:** 1909-05-01

**Authors:** 


### Anæsthetics.

### THE PERCENTAGE ADMINISTRATION OF CHLOROFORM.

It is more than six years since Professor Waller first seriously called the attention of the medical profession and of anæsthetists to a densimetric method of determining the percentage of chloroform vapour in the air supplied to patients or animals during anæsthesia. At that time the contention that the rule of thumb methods thitherto, and still now, in vogue possess grave disadvantages was quite a novel one, and one which practising anæsthetists were disinclined to admit. There is no doubt that a good deal of their scepticism was based on the fact that the apparatus used by the eminent physiologist in certain demonstrations at London hospitals was clumsy, cumbersome, and not very efficient. On the other hand, the physiologists were able to retort with figures showing the dangers of chloroform administration by the old methods, though perhaps it was not always emphasised sufficiently how greatly this is reduced in skilled hands. At all events there was a certain division of opinion between physiologists and anæsthetists, which was largely due to the different standpoints of pure science and of commonplace expediency from which respectively the question was viewed.

Matters, however, have progressed since then; the latest contribution to the literature of the subject of percentage administration comes from the pen of a physiologist, and is as fully informed with practical suggestions as the most strictly empirical anæsthetist could desire. Dr. N. H. Alcock* has devoted much time and trouble to the investigation of the trustworthiness of an apparatus of which he published a description last year, and of the lessons which he, in conjunction with professional anæsthetists, has learned from its use.

He starts by pointing out that he makes as yet no attempt to measure the quantity of chloroform absorbed or to trace its fate in the body. The percentage of vapour in the inspired air is, as he remarks, the only factor under the absolute control of the administrator, and so for the present he confines himself to that. All machines for offering known strengths of vapour for inhalation are based upon one of two principles: either the inspiratory act of the patient draws air through a chamber containing chloroform, or else the air is propelled on a "plenum" system by artificial means to a closed space which includes the external organs of respiration of the patient. Dr. Alcock uses the latter system, as he believes there is less risk of obstruction to a free airway.

He first deals with the errors of method possible with his machine, and believes they amount at the outside to 0.1 per cent. The errors of the actual apparatus itself seldom also exceed this, but may occasionally reach 0.2 per cent. Then he determines whether variations in the quantity of air driven through the chloroform chamber have any effect on the percentage of the drug taken up, and he finds that between 20 and 8.5 litres per minute they have none. The usual supply is 16 to 20 litres. The author then proceeds to consider the application of his apparatus in actual administrations by the St. Mary's Hospital anæsthetists. He plots out the details of each case by a graphic method which is both lucid and ingenious. Along the ordinate of his curves he marks a scale which shows the percentage of chloroform delivered, while the abscissa is divided into minutes of time from the commencement of the operation. The curves thus constructed show the rapid rise in the strength of the vapour offered, from nil to 2 per cent. (usually) at the end of two minutes by regular increments of a quarter per cent. every fifteen seconds. After this the rise is made more gradual, and is adjusted according to the needs of the individual case. Sometimes it is necessary to offer 3.5 per cent., the most which Dr. Alcock's apparatus allows; and in rare cases even this is not enough, but resort has been made to mask and drop bottle. In children, as is to be expected, less suffices, and 2 per cent. is as a rule enough, though to this exceptions occur. Stout people take more than thin, and alcoholic subjects more than all, as is well known. Individual peculiarities were also disclosed, not to be accounted for by any known factor. This again is a very old story.

The induction period is stated to be commonly eight to ten minutes. When induction is complete, 2 per cent. is generally ample, and this may be cut down to 1.5 in a few minutes, and to 1 per cent. within half an hour in most cases. The charts of actual administrations show very well what is called by Mr. R. Gill the law of diminishing resistance, the curve approaching the abscissa as the ordinate of vapour strength diminishes. Thus one patient was kept perfectly anæsthetised for two and a quarter hours, during the last ninety minutes of which time but 0.75 per cent. of chloroform was being supplied in the inspired air.

Another important lesson deduced, though here again it is a truth with which experienced anæsthetists are already familiar, is that the effects of overdose are not seen at once, but require about a minute for their development. When the mechanism of respiration and gaseous interchange in the lungs, and of absorption and distribution by the blood stream are considered, there is little in this to be surprised at. The practical point is more than ever brought home that incessant vigilance to detect the very faintest and earliest sign of overdosage is ever called for from an anæsthetist. Thus only can he take in time the measures necessary for the protection of his patient.

Dr. Alcock, after watching various anæsthetists at work with a mask and drop bottle, suggests that in most cases a somewhat stronger vapour than what he considers the optimum is delivered during the first two minutes, and a somewhat weaker one during the rest of the induction. A first-rate anæsthetist with a drop bottle is probably as reliable as with any mechanical apparatus.

* *British Medical Journal*, February 6, 1909, p. 325.

